# Green and scalable synthesis of nanocrystalline kuramite

**DOI:** 10.3762/bjnano.10.202

**Published:** 2019-10-29

**Authors:** Andrea Giaccherini, Giuseppe Cucinotta, Stefano Martinuzzi, Enrico Berretti, Werner Oberhauser, Alessandro Lavacchi, Giovanni Orazio Lepore, Giordano Montegrossi, Maurizio Romanelli, Antonio De Luca, Massimo Innocenti, Vanni Moggi Cecchi, Matteo Mannini, Antonella Buccianti, Francesco Di Benedetto

**Affiliations:** 1 Department of Earth Sciences, University of Florence, Via La Pira 4, 50121 Firenze, Italy; 2 Department of Industrial Engineering, University of Florence, Via di S. Marta 3, 50139 Firenze, Italy; 3 INSTM Florence Research Unit, Via Lastruccia 3-13, 50019 Sesto Fiorentino (FI), Italy; 4 Department of Chemistry, University of Florence, Via Lastruccia 3-13, 50019 Sesto Fiorentino (FI), Italy; 5 ICCOM-CNR, Via Madonna del Piano 10, 50019 Sesto Fiorentino (FI), Italy; 6 IOM-CNR, c/o European Synchrotron Radiation Facility 71, avenue des Martyrs, 38043 Grenoble Cedex 9, France; 7 IGG-CNR, Via La Pira 4, 50121 Firenze, Italy,; 8 Museo di Storia Naturale, Sistema Museale di Ateneo dell’Università degli Studi di Firenze, Via La Pira 4, 50121 Firenze, Italy

**Keywords:** Cu_2_ZnSnS_4_ (CZTS), Cu_3_SnS_4_ (CTS), green chemistry, kuramite, photovoltaic materials, solvothermal synthesis

## Abstract

The new generation of solar cells aims to overcome many of the issues created by silicon-based devices (e.g., decommissioning, flexibility and high-energy production costs). Due to the scarcity of the resources involved in the process and the need for the reduction of potential pollution, a greener approach to solar cell material production is required. Among others, the solvothermal approach for the synthesis of nanocrystalline Cu–Sn–S (CTS) materials fulfils all of these requirements. The material constraints must be considered, not only for the final product, but for the whole production process. Most works reporting the successful synthesis of CTS have employed surfactants, high pressure or noxious solvents. In this paper, we demonstrate the synthesis of nanocrystalline kuramite by means of a simpler, greener and scalable solvothermal synthesis. We exploited a multianalytical characterization approach (X-ray diffraction, extended X-ray absorption fine structure, field emission scanning electron microscopy, Raman spectroscopy and electronic microprobe analysis (EMPA)) to discriminate kuramite from other closely related polymorphs. Moreover, we confirmed the presence of structural defects due to a relevant antisite population.

## Introduction

In the last decade, advances in materials science and nanoscience have led to the development of new solar cell technologies. Today, they hold promise to overcome the environmental issues created by silicon-based devices. Such devices are difficult to decommission, their production requires energy-hungry processes and they are not compatible with flexible devices. For example, materials such as copper indium gallium selenide (CIGS) and perovskite materials have raised several concerns. They contain rare/scarce raw materials and involve production and decommissioning processes which are environmentally serious [1–8]. Thin films of kesterite and kuramite (tetragonal Cu_2_ZnSnS_4_ and Cu_3_SnS_4_, respectively) are among the most sustainable third generation solar cell technology materials [9–11]. Their best conversion efficiencies span from 12.6% (using Cu_2_ZnSnS_4−_*_x_*Se*_x_* obtained with a hydrazine-assisted solution process [12–13]) to 10.6% (using a vacuum process [14]) and 8% (obtained with an electrochemical process [15]). These multinary sulfides have extremely low toxicity, consist of earth-abundant elements [3] and can be obtained by means of processes that are more energy efficient with respect to those used for silicon-based solar cell production [2,16–27]. Most of these processes yield nanocrystalline materials that can be easily dispersed in inks [28–33], leading to a plethora of new applications, even processing on flexible surfaces. Most of the efforts in this research field aim at the optimal trade-off between the solar energy conversion performance, scalability and sustainability of these nanocrystalline multinary sulfides [34–35]. To this end, researchers have produced many different polymorphic and isomorphic phases related to kuramite. Indeed, the Cu–Sn–S pseudo ternary compositional field presents several closely related phases: mohite (monoclinic Cu_2_SnS_3_) with its tetragonal and cubic polymorphs [36–37], kuramite (Cu_3_SnS_4_) [38–40], Wang’s phase (Cu_4_SnS_4_) [41–42], and Cu_4_Sn_7_S_16_ [43], among others. Natural ternary Cu–Sn–S phases populate the pseudo ternary compositional field mostly along the two CuS–SnS and Cu_2_S–SnS_2_ joints [36,44]. Their nanocrystalline counterparts have broadened X-ray diffraction peaks. This limits the discrimination of the different phases and the study of their possible solid solutions [45]. Due to such difficulties in phase attribution, hereafter, we will refer to a generic Cu–Sn–S ternary compound as CTS. In this study, we present an easily scalable solvothermal synthesis, representing a simple, green and room-pressure method to obtain nanocrystalline kuramite. We synthesized three samples using a solvothermal approach, which was carried out under mild conditions in ethylene glycol as a green solvent. We tackled the aforementioned difficulties in phase assignment by means of thorough characterization, including X-ray diffraction (XRD), scanning electron microscopy (SEM), principal component analysis (PCA) of the wavelength dispersion spectroscopy (WDS) data, X-ray absorption spectroscopy (XAS) and Raman spectroscopy.

### Materials and Methods

#### Synthesis

The reactants necessary for the three syntheses are: CuCl_2_·2H_2_O (Merck), ZnCl_2_ (Merck), SnCl_2_·2H_2_O (Riedel-de Haën AG), thiourea SC(NH_2_)_2_ (TU, Merck), and ethylene glycol (EG, 99%, Alfa Aesar). The solvothermal approach used in this work exploits TU as a sulfide source in a one-pot synthesis with the solvent and the metal salts similar to the synthesis of FeS_2_ and Cu_2_ZnSnS_4_ [35,45] nanopowders. In a two-neck flask we mixed appropriate amounts of metal chlorides and TU with EG. The synthesis proceeded with the formation of a dark precipitate under reflux for a reaction time of 2 h. We collected the precipitates after having let the flask cool down to room temperature. We centrifuged and washed the products at least five times in absolute ethanol and dispersed the particles by means of sonication. Finally, the powders were dried under air at room temperature. Three different syntheses are presented in this study using different precursor concentrations. The mixing of the chemicals was performed in a 100 mL flask filled with 50 mL of EG (Table 1). Sample S1 is a Zn-containing kuramite test sample that was previously characterized in another paper [45].

**Table 1:** Molar amounts of precursors dissolved in 50 mL of ethylene glycol (EG).

	CuCl_2_·2H_2_O (mmol)	ZnCl_2_ (mmol)	SnCl_2_·2H_2_O (mmol)	SC(NH_2_)_2_ (mmol)	Ref.

S1	0.8	0.4	0.4	1.6	[45]
S2	1.2	–	0.4	1.6	this study
S3	0.8	–	0.4	1.6	this study

#### Characterization

A thorough description of the experimental procedures is reported in the Section 1 of Supporting Information File 1. We acquired the SEM micrographs of the powders deposited on an aluminum stub after coating the sample with Au. The phase determination of the synthetic products was carried out on the Rietveld refinement of the XRD pattern by using the “GSAS II” software [46]. The instrumental parameters for the Rietveld refinement have been set to the values reported elsewhere [47]. The chemical element compositions of the samples were analyzed through an electron microprobe. The data were corrected using the Pouchou and Pichoir (PAP) matrix correction [48–49]. We exploited a CuS (covellite) standard to better estimate the Cu/S ratio [45]. The PCA procedure required the transformation of the experimental data by using the log-centered transformation [50–52] to achieve a robust and unbiased analysis. On this basis, we performed a partition of the elemental concentrations dwelled on a variance decreasing criteria [53–55]. Raman spectroscopy was performed with a He–Ne laser source emitting at 632.8 nm with a laser spot on the sample of about 10 μm^2^. The main reference for the positions of the Raman peaks is from the RRUFF database [56]. XAS measurements at Cu and Sn K-edge (8978.9 and 29200.1 eV, respectively) were carried out at the LISA CRG beamline (BM-08; [57]) at the European Synchrotron Radiation Facility (ESRF, Grenoble, France). The software ATHENA [58] was used to average multiple spectra. Standard procedures [59] were followed to extract the structural extended X-ray absorption fine structure (EXAFS) signal. EXAFS spectra were fitted through the use of ARTEMIS, FEFF8 and ATOMS software in the Fourier transform (FT) space [58,60–61].

## Results

### Scanning electron microscopy

**Figure 1:**
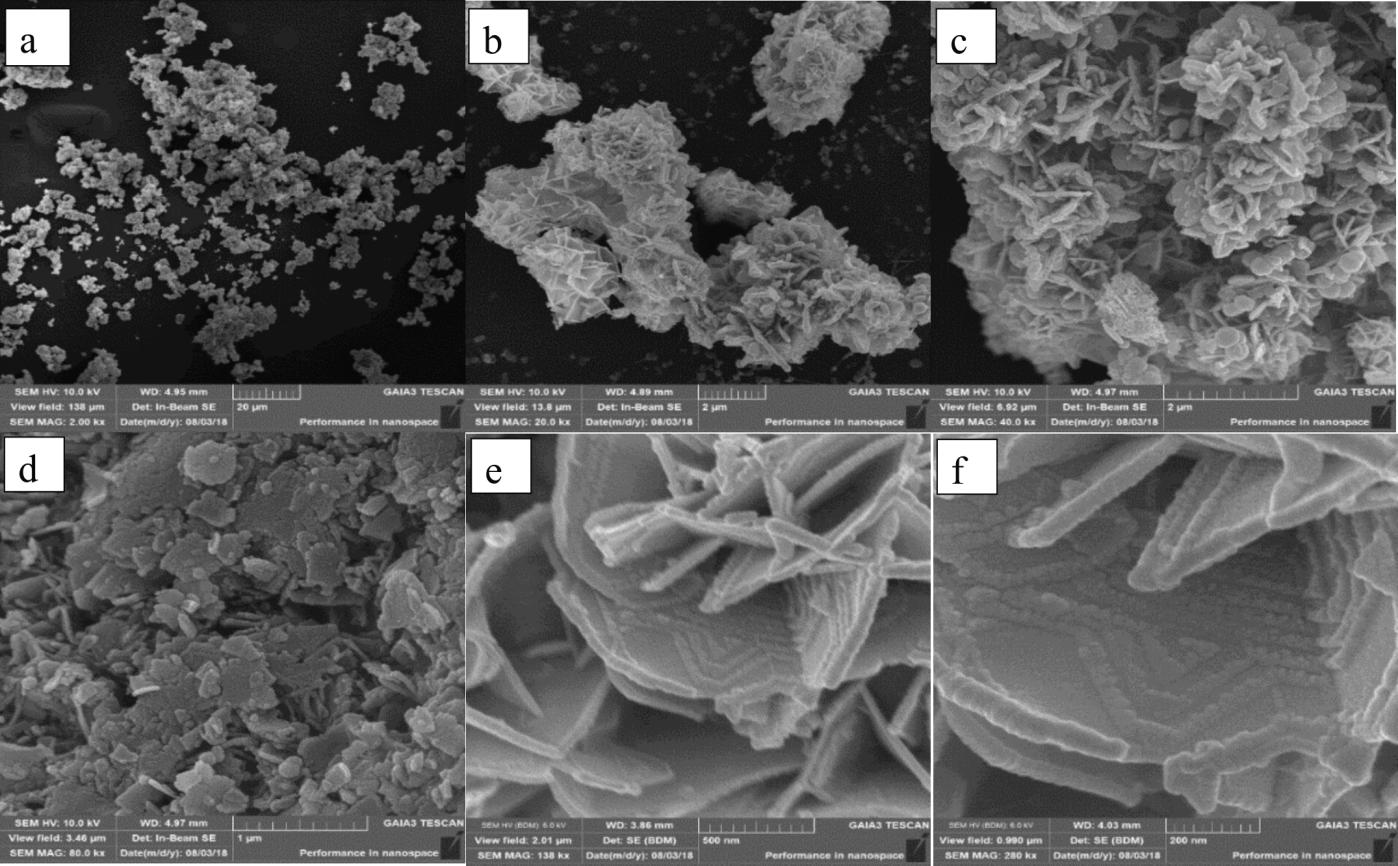
SEM micrographs (a) 2000× sample S3, (b) 20,000× sample S1, (c) 40,000× sample S3, (d) 80,000× sample S3, (e) 140,000× sample S1 and (f) 280,000× sample S1.

Typical SEM images are presented in Figure 1 where no appreciable differences among the tens of observations performed on different samples was found. Figure 1a shows an overview of the powder agglomerates at 2000× where the main features of the micromorphology are already visible. Figure 1b (20,000×) shows that the powder is mainly constituted by intergrown platelets with different orientations. Most of the platelets in Figure 1c (40,000×) were 25–100 nm thick and 1–3 μm wide. Figure 1d (80,000×) shows the presence of an additional distribution of platelets much smaller and more randomly distributed than the one observed in Figure 1c. Further magnification (Figure 1e,f) enables the observation of regular shapes (probably enhanced by the Au coating) ascribed to surficial structural defects of the platelets or to a new growing facet.

### Electronic microprobe analysis (EMPA)/wavelength dispersion spectroscopy (WDS)

**Figure 2:**
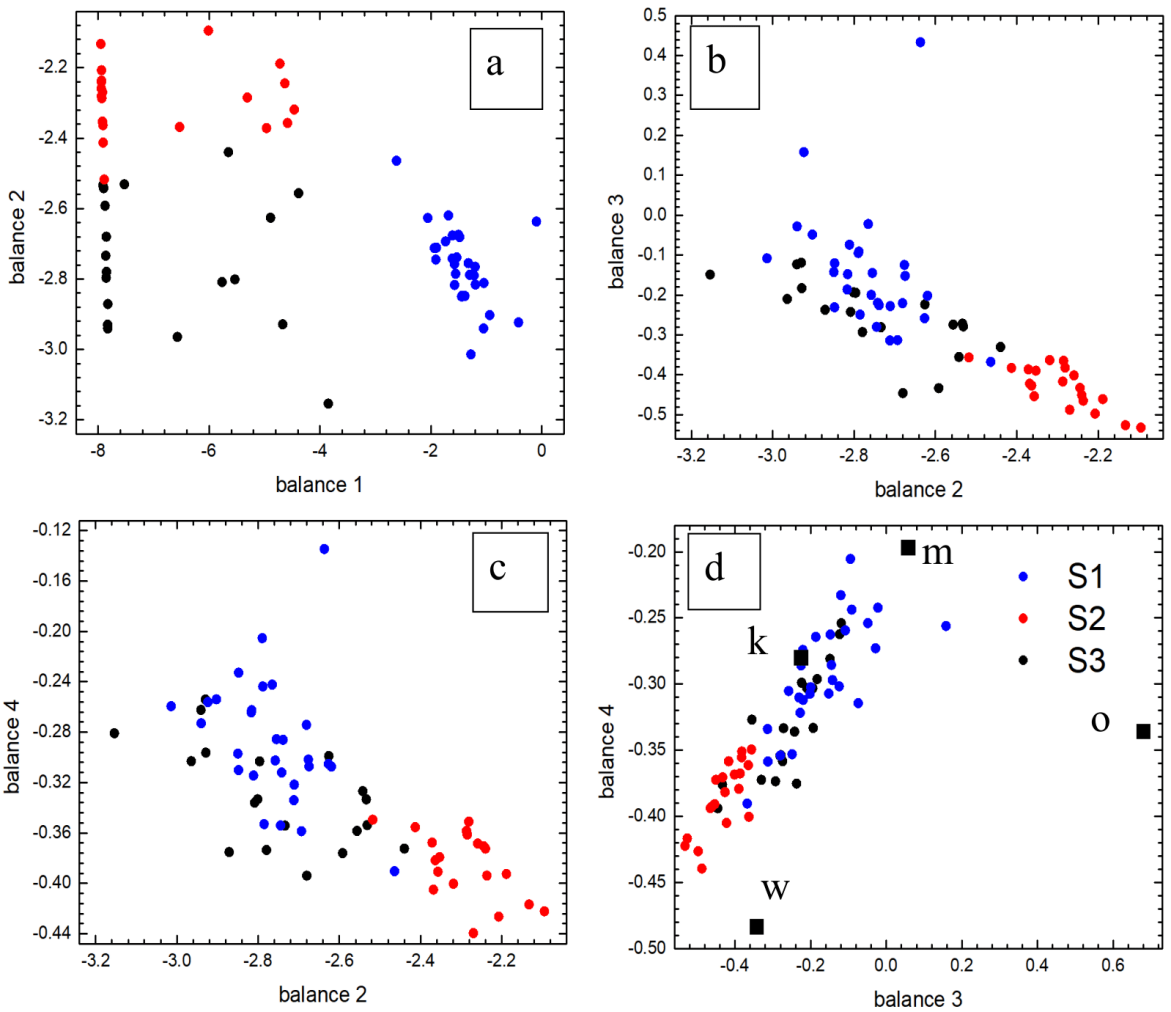
Plot of the trends between two difference balances, where the squares represent the position of the considered phases in the transformed space k) Cu_3_SnS_4_, m) Cu_2_SnS_3_, w) Cu_4_SnS_4_ and o) Cu_4_Sn_7_S_16_. Sample S1, S2 and S3 compositions are indicated by blue, red and black dots respectively.

EMPA analysis showed and confirmed that the molar ratios between Cu, Zn, Sn, S and Cl in the sample does not correspond to the reaction mixture. The initial assessment of the dataset variability has been carried out through the use of the biplot (Figure S1, Supporting Information File 1) methodology for compositional data analysis [51,62]. This analysis suggests a strong segregation between Sn and Cl as well as a partial segregation between Cu and S. Since the variability of Cu and S is much smaller than Sn and Cl, further inspection is required. For this purpose, we applied the partition criteria based on the decreasing variance. This enables the reduction of the five dimensions of the original compositional space, leading to the four different balances shown in Table 2. Figure 2 presents the most relevant relationship between these balances in the different samples. There is nearly no dependence between balances 2 and 1 (Figure 2a) for any of the samples, suggesting that Zn and Cl are segregated in associated phases (unreacted species) for sample S2. Since there is an inverse relationship between balances 3 and 2, as well as between balances 4 and 2, (Figure 2b,c) Cl is assumed to be in a phase separated by Sn and S. Indeed, the balances 4 and 3 are directly correlated, which reveals that Sn and S are colocalized (Figure 2d). Hence, Cu is the only cation without an inverse relationship with Cl, suggesting that Cu and Cl must be partially colocalized in an associated phase. Figure 2d also shows that the data points of samples S2 and S1 cluster around the position of kuramite in the transformed space, suggesting that samples S1 and S3 are homogeneously consistent with the composition of kuramite. The formula units of the samples were calculated after removing the outlying data point [52] and not considering the percentage of Cl, since the statistical analysis reveals that it is mostly located in an associated phase. Two factors should be considered: 1) the difference between the atomic percentage of Cu and S in the CuS standard is systematically close to 3%; 2) the segregation of Cl in an associated phase exclusively with Cu. Conversely, Sn and S are present in the main phase with the highest proportion of Cu. On this basis, we were able to correct the result of the EMPA analysis by: 1) adding 1.5% to the atomic percentage of the S atomic percentage and subtracting 1.5% from the Cu atomic percentage and 2) subtracting an amount of CuCl_2_ from the analysis, such as to account for the total amount of Cl.

**Table 2:** Result of the isometric log-ratio analysis on the basis of the decreasing of the variance criteria. When the denominator is characterized by more than one variable, it is represented by the geometric mean of the involved elements.

Label	Definition of the balance	Variance

balance 1	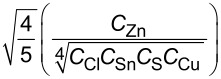	8.2066
balance 2	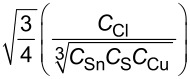	0.0629
balance 3	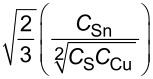	0.0269
balance 4	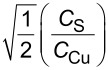	0.0034

The formula units of the three phases and of the phases statistically close to the three samples (Figure 2d) are normalized to 8 apfu (atoms per formula unit). These further elaborations result in an agreement with kuramite’s reference composition (Table 3). Sample S1 matches the composition of a solid solution in the kesterite–kuramite joint (15.6% kesterite). Sample S3 is consistent with the composition of kuramite in the range of 1σ. Sample S2 has to be considered carefully due to the highest presence of Cl in associated phases and the possible presence of other Cu- and S-bearing associated phases. Still, the compositional data of the final products require further consideration after a discussion of the results obtained with other characterization techniques.

**Table 3:** Recalculated composition of samples S1, S2 and S3 after subtraction of the estimated amount of CuCl_2_. Comparison with the raw composition (apfu (atoms per formula unit) (raw)) and the composition of the statistically close phases.

	apfu (raw)	apfu	Mohite	Kuramite	Wang

S3					

S	4.01(6)	4.09(6)	4.00	4.00	3.56
Cu	3.02(6)	2.92(6)	2.67	3.00	3.56
Sn	0.97(2)	0.99(2)	1.33	1.00	0.889

S2					

S	3.96(4)	4.02(4)	4.00	4.00	3.56
Cu	3.23(4)	3.15(4)	2.67	3.00	3.56
Sn	0.81(1)	0.81(1)	1.33	1.00	0.889

S1					

S	3.97(8)	3.97(8)	4.00	4.00	3.56
Cu	2.89(6)	2.89(6)	2.67	3.00	3.56
Zn	0.156(8)	0.157(8)	–	–	–
Sn	0.98(2)	0.98(2)	1.33	1.00	0.889

### X-ray diffraction

**Figure 3:**
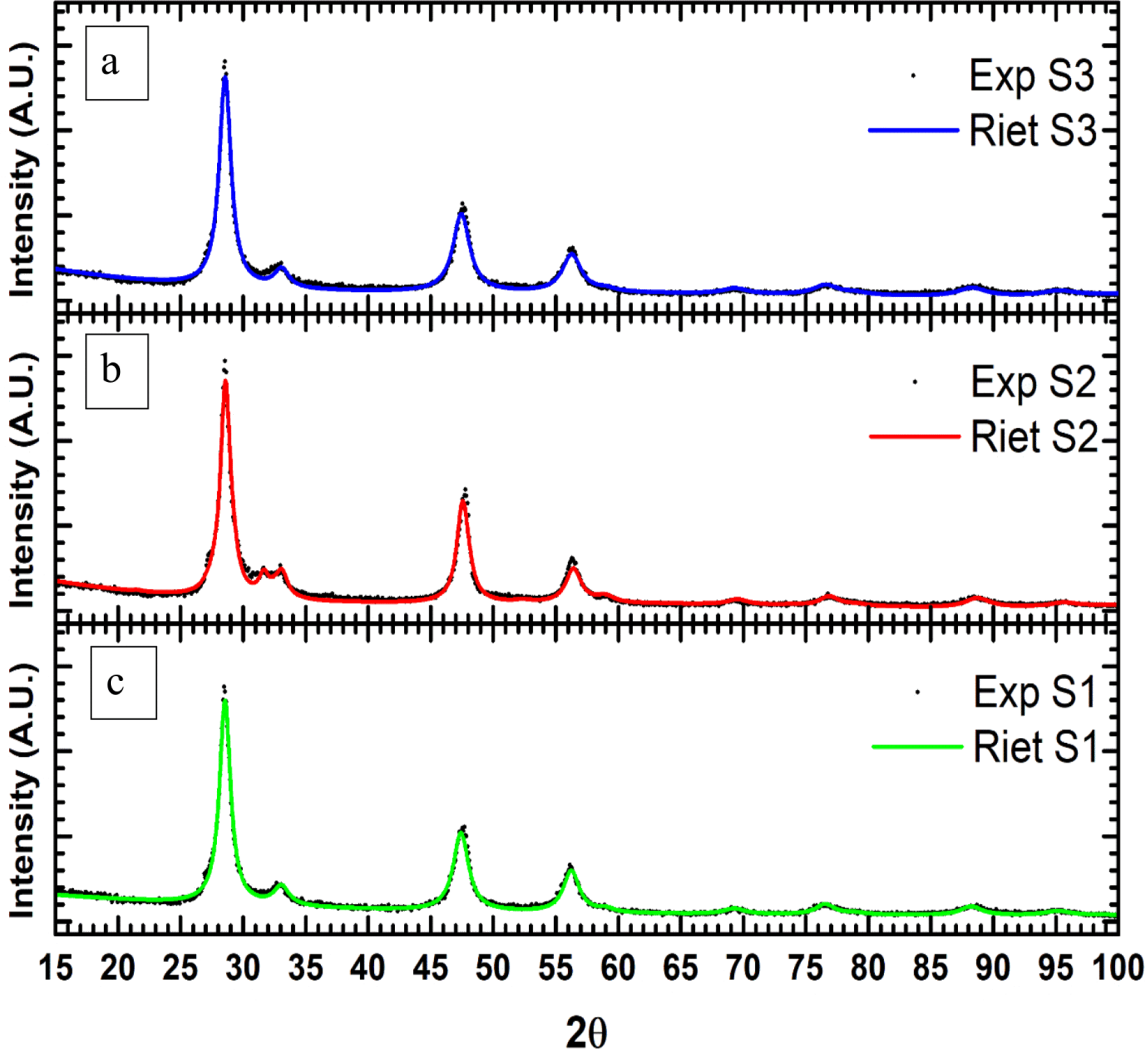
Experimental and calculated diffractograms and related models for sample a) S3, b) S2 and c) S1. Exp and Riet are the experimental data and the Rietveld refinement, respectively.

We performed a Rietveld refinement of the experimental patterns in the framework of the tetragonal CTS structural model (Figure 3) excluding a substantial presence of Wang’s phase [63]. The refined lattice parameters (see Table S1, Supporting Information File 1) compare very well with those of kuramite (*a* = 5.445 Å; *c* = 10.75 Å; JCPDS 33-0501 [64]) or mohite (ICDD PDF 04-010-5719; *a* = 5.413 Å; *c* = 10.82 Å [43]). In agreement with the discussion reported elsewhere [45], the diffraction pattern of sample S1 can be refined with a kuramite structural model due to its nearly absent Zn content and very similar atomic structural factors of Cu^2+^, Cu^+^ and Zn^2+^ [65]. A refinement can be attempted also by assuming a cubic CTS model based on the sphalerite-like structural model (JCPDS 5-0566 [66]). In this model, we derived the cubic cell from the tetragonal supercell, reducing it to its cubic subunit and assuming a complete disordering of the cation sites (see Figure S3, Supporting Information File 1).

The cubic structural model yields better results even for a lower number of free parameters (Tables S1 and S2, Supporting Information File 1): namely, three Debye–Waller factors for the cubic CTS and four for the tetragonal CTS. Only the refinement of sample S2 required an associated phase (covellite, JCPDS 06-0464 [67]). The weight fractions of covellite for the tetragonal and cubic models of the CTS phase are 23% and 28%, respectively. For the cubic structural model, the unit cell for the S3 sample shows a definite lower discrepancy with respect to kuramite than to mohite, while for samples S2 and S1 the discrepancies are lower for mohite. For the tetragonal structural model, the unit cell volumes of the three samples are very close to the volume of the unit cell of mohite. However, it should be noted that for all the samples the refined tetragonal cell would appear very distorted with respect to the tetragonal mohite cell (e.g., for sample S1 elongated by 1% along the *c*-axis and contracted by 0.5% along the *a*- and *b*-axis), while the difference with respect to the kuramite structure tends to the contraction of the three cell parameters. As a result, the cell would be smaller in volume (discrepancy of 0.5%) but not distorted. For the three samples the average crystallite size is in the range between 9 and 13 nm with a slight difference between the two structural models of the CTS phase (Tables S1 and S2, Supporting Information File 1). It is worth mentioning that the crystallite size is smaller than the average size of the features/particles identified in the FESEM investigations. This is in line with our previous reports [68] on the TEM-detected crystallite size of kuramite nanopowder obtained via a similar solvothermal approach and with recent observations on kesterite synthesized by means of the same approach exploited in this study [45]. No preferential orientation can be established (see Supporting Information File 1, Section 2.2).

### Raman spectroscopy

**Figure 4:**
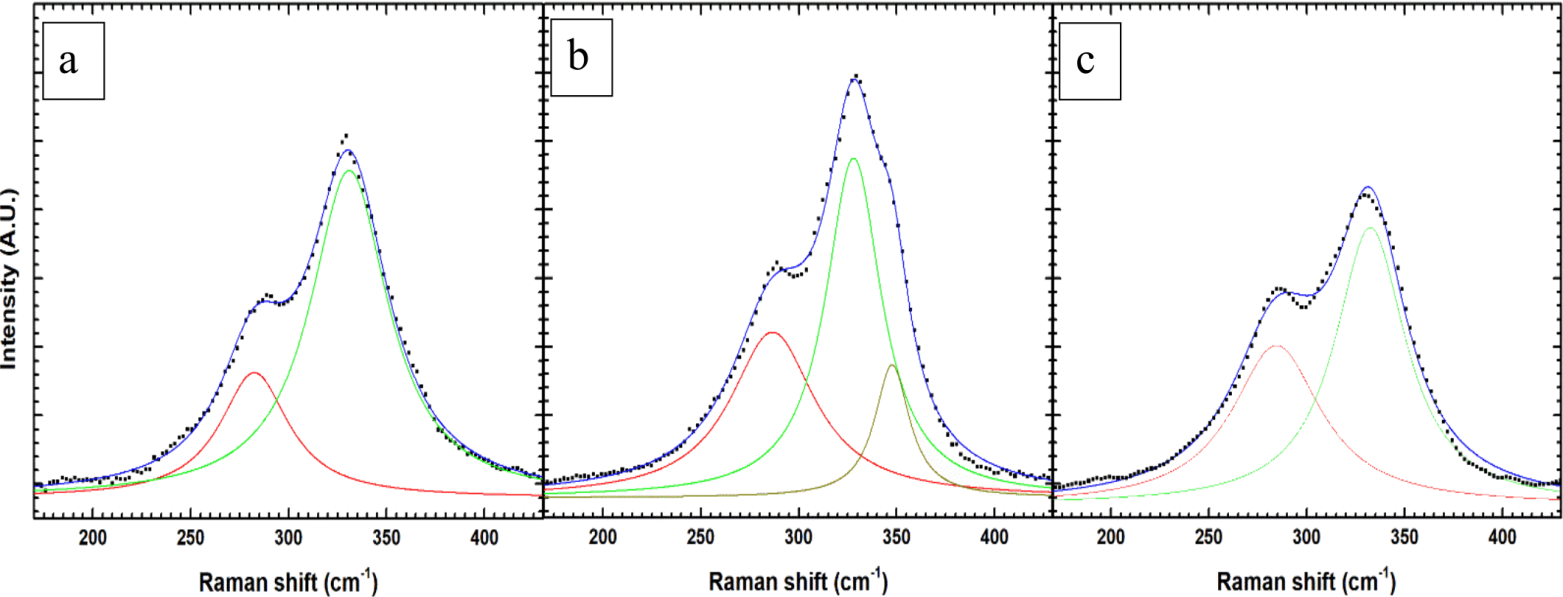
Raman spectra for the samples a) S1, b) S2 and c) S3.

The details of the averaged Raman spectra in the range 165–430 cm^−1^ are shown in Figure 4 for the samples. There are at least two evident maxima for each sample, but sample S2 shows an additional very pronounced shoulder at 350 cm^−1^. Figure 4a,c shows the results of a nonlinear fitting process carried out with two Lorentzian peaks for samples S1 and S3 (adding further Lorentzian components to the spectra did not lead to an reduction in the R factor). Conversely, adding a third Lorentzian component to the sample analysis of sample S2 led to a reduction of the R factor of 0.6%. The results of the fits show that the widths of the Lorentzian profiles are clearly wider than the widths found in the literature [11,30,69–70]. These values are related to size-induced line broadening typical of nanocrystalline samples. Indeed, they compare well with other nanocrystalline synthetic tetragonal CTS samples [30,69,71] (See Table S3, Supporting Information File 1). Such width values impair the fit of other contributions in the Raman spectra, the details of which are presented in Table S3, Supporting Information File 1. Moreover, there is no clear evidence of other associated phases than those already discussed. The positions of the first and second components are consistent with those elsewhere assigned to kesterite (RUFF ID: R120098.2 [72–73]) or stannite (RUFF ID: R050187 [74–75]). Concerning the differences in position between the first and second components for the samples S1 and S2, they have been found to be equal and in the range of 1σ. These values compare well with the difference expected from the peaks assigned to the structurally similar tetragonal kesterite [72] and stannite [75–76], suggesting that the main phase of these three samples is structurally related to the latter. In other studies, such difference have been assigned to tetragonal kuramite [70] or mohite [71]. The ratio between the areas of the first and second components is roughly 2.9(1), 1.35(7) and 1.38(7) for samples S1, S2 and S3, respectively: this points out that the main phases in samples S3 and S2 are very similar, and only a slight difference can be found for sample S1, which is consistent with the Raman spectra on the kesterite-type compounds [72–73]. The small shoulder at 350 cm^−1^ in the spectrum of sample S2 has no straightforward attribution (see Supporting Information File 1, Section 2.3 for further information). The decomposition of the reducible representations of the Γ point in the Brillouin zone [77–79] yields three degenerated normal modes that are active both in IR and Raman spectroscopy with *irrep*** Γ_5_** = **T_2_** for the *F*−43*m* space group and five normal modes (with *irrep* **Γ_1_**, **Γ_3_**, **Γ_4_**, **Γ_5_**) for the *I*−42*m* space group (Table S4, Supporting Information File 1). Kuramite usually presents more Raman peaks in the same range of Raman of the spectra presented (165–430 cm^−1^) [75–76]. Indeed, since the Raman spectra of our samples showed two wide peaks, they are probably due to the overlap of the five Raman peaks expected for the *I*−42*m* space group. On the contrary, a cubic structure would have had only one peak. This is predicted by the vibrational mode analysis and confirmed by the Raman spectra of sphalerite showing only one sharp and very intense peak (RUFF ID: R040136; [80–81]).

### X-ray absorption spectroscopy

**Figure 5:**
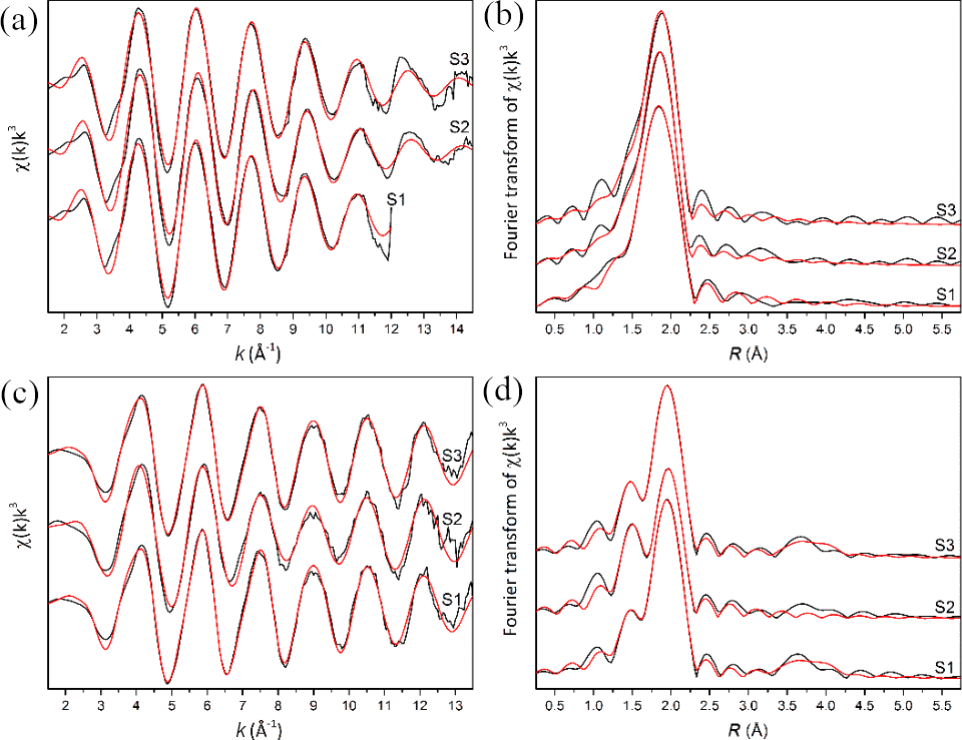
Cu (a,b) and Sn (c,d) K-edge EXAFS (a,c) and Fourier transform (b,c) of the studied samples. The experimental data are reported as black lines and the red lines represent the fits.

EXAFS (at Cu and Sn K-edge) of samples S1, S2 and S3, along with the respective Fourier transforms, are shown in Figure 5 together with the corresponding multiparameter fits. The fit results for the I coordination shell with S are reported in Table 4. EXAFS analysis at the Cu K-edge leads to Cu–S bond distances extremely close to those of Cu in a CTS structure, in the typical range of a four-fold coordination with S (e.g., the work of Bacewicz et al. [82] and references therein); no second shell signal is observable, which is in reasonable agreement with the lower ordering expected from the extremely small crystallites (e.g., in the work of Giaccherini et al. [45]). Data from Sn K-edge indicate a first shell with bond distances compatible with Sn in tetrahedral coordination with S atoms in a CTS structure. Although the EXAFS signal is characterized by an almost single-frequency oscillation, the fitting procedure with the sole Sn–S path leads to a residual signal with a well-defined oscillation period. Most of the residual signal was successfully fitted by adding a second Sn–O path that took into account a partial oxidation of the samples, clearly visible as a shoulder on the I shell peak in the Fourier transform space. However, a small residual signal was still visible, corresponding to the small bump at ≈3.7 Å in the Fourier transform space. This residual could be fitted in samples S1 and S3 as a mixed Cu/Sn II shell with Cu and Sn atoms at ≈3.82(2) Å and 4.01(2) Å, respectively. These results are in agreement with Sn in a CTS structure with a high degree of disorder between Cu and Sn.

**Table 4:** EXAFS multiparameter fits for the studied samples.

	Cu K edge				Sn K edge			
		
	*S*_0_^2^	shell	*R* (Å)	σ^2^ (Å^−2^)	*S*_0_^2^	shell	*R* (Å)	σ^2^ (Å^−2^)

S1	0.70(5)	4S	2.283(6)	0.0083(8)	1.14(6)	4S	2.407(5)	0.0069(4)
S2	0.71(5)	4S	2.270(5)	0.0084(6)	1.1(1)	4S	2.402(5)	0.001(2)
S3	0.69(6)	4S	2.284(7)	0.0077(8)	1.13(6)	4S	2.404(5)	0.0071(5)

## Discussion

The morphological analysis showed that all the samples appear very similar when compared with one another and their lamellar aspect is consistent with previous reports [35,45]. The Rietveld refinement and the analysis of the Raman spectra confirm the result of the compositional analysis and supports the conclusion that kuramite is the main phase. A large increase of the crystalline size and defective strain is responsible for the significant line broadening of the diffraction peaks. Still, no prevalent amorphous phase can be observed in the diffractograms. On this basis, the XRD results support the mixed occupancy of the cations in the available tetrahedral sites, which are suggested to play a decisive role in the semiconducting and transport properties of the compound [2,33,82]. It should be noted that for both kuramite and mohite the cubic polymorphs have been reported but only at high temperatures of 680 °C and 780 °C, respectively. We must take into account that at room temperature they could be metastable phases. These results compare well with the EXAFS spectra at the Cu and Sn K-edge showing that the local structure around Cu and Sn atoms is compatible with a CTS structure. Still, the spectra at both the K-edges show a marked dampening of the second shell signal. This revealed significant differences between the Sn and Cu local environments, which is probably due to the small crystallite size and the high concentration of defects (in agreement with the XRD broadening). The presence of a second coordination shell with both Cu and Sn atoms is observable only at the Sn K edge. This suggests that Sn is completely segregated in a CTS phase with a mixed occupancy at the tetrahedral sites (as suggested also by the PCA results). On the contrary, we cannot exclude that Cu may be present in an amorphous Cu(I) sulfide with Cu–S first shell distances compatible with a CTS structure. The Raman spectra of the samples show the presence of two wide peaks ascribed to tetragonal Cu_3_SnS_4_. Hence, we can exclude that the short- and long-range disorder reckoned by both EXAFS and XRD lead to the increasing symmetry with respect to the natural counterparts (*I*−42*m → F*−43*m*) due to the complete mixing of Sn and Cu sites. The presence of associated phases such as SnS, Sn_2_S_3_ and Cu_2_SnS_3_ could be excluded (samples S1 and S3) [71]. The statistical analysis of the EMPA compositional data clearly showed that Zn is present only in sample S1, although this is very difficult to detect with EDX [45], and it confirmed that the composition of sample S3 is consistent with kuramite’s composition. We found that the final product is unaffected by the addition of ZnCl_2_ in the batch of the reactants. Comparing the products obtained by the pure kuramite batches (samples S2 and S3) with those obtained by the S1 test sample suggests that no effect on the crystalline structure is detectable. Conversely, the only apparent effect is to reduce the Cu/Sn ratio in the reacting mixture. We can perform a further elaboration of the composition of sample S2. If the amount of covellite (28%, as calculated by Rietveld refinement) is removed from the composition, the data of sample S2 fit better with that of kuramite within 3–4σ (Table S5, Supporting Information File 1).

## Conclusion

In this paper, we report the successful synthesis of nanocrystalline kuramite by a simple, green and scalable one-pot approach without the use of surfactants. Due to the complex crystal chemistry of the phases in the Cu–Sn–S system, we employed a deep structural and compositional analysis to correctly assign the CTS phases. We emphasize that a multianalytical approach, similar to that performed here, is necessary to gain a deeper understanding of the phase and chemical sample composition. On this basis, we were able to assign kuramite as the main phase in each sample with only a minor presence of associated phases in sample S3. We showed that to limit the amount of covellite in the final product, a relevant factor is the molar defect of Cu precursor in the EG. The crystal chemical analysis (by Raman, XRD and EXAFS) revealed a random distribution of antisite defects, Sn_Cu_ and Cu_Sn_, in the tetragonal structure while still maintaining the *I*−42*m* symmetry. These chemical and crystal-chemical considerations suggest a complex chemistry for ethylene glycol based solvothermal synthesis (as elsewhere discussed for kesterite [45]). Further studies should be undertaken to relate the conversion properties of these materials to the antisite population.

## Supporting Information

File 1 Additional information regarding the synthesis and characterization of the samples.
