# Supplementary material for: Green and scalable synthesis of nanocrystalline kuramite
Source: Beilstein J Nanotechnol. 2019 Oct 29;10:2073–83. doi: 10.3762/bjnano.10.202 (PMC6839557; doi:10.3762/bjnano.10.202)
Supplement: File 1 — Additional information regarding the synthesis and characterization of the samples. [file Beilstein_J_Nanotechnol-10-2073-s001.pdf]

BEILSTEIN JOURNAL OF NANOTECHNOLOGY

Supporting Information

for

Green and scalable synthesis of nanocrystalline kuramite

Andrea Giaccherini, Giuseppe Cucinotta, Stefano Martinuzzi, Enrico Berretti, Werner Oberhauser, Alessandro Lavacchi, Giovanni Orazio Lepore, Giordano Montegrossi, Maurizio Romanelli, Antonio De Luca, Massimo Innocenti, Vanni Moggi Cecchi, Matteo Mannini, Antonella Buccianti and Francesco Di Benedetto

Beilstein J. Nanotechnol. **2019,** *10,* 2073–2083. doi:10.3762/bjnano.10.202

Additional information regarding the synthesis and characterization of the samples

License and Terms: This is a supporting information file under the terms of the Creative Commons Attribution License (http://creativecommons.org/licenses/by/4.0). Please note that the reuse, redistribution and reproduction in particular requires that the authors and source are credited.
The license is subject to the *Beilstein Journal of Nanotechnology* terms and conditions: (https://www.beilstein-journals.org/bjnano)

Section 1: Materials and methods

1.1 SEM

We acquired the SEM micrographs by using a Tescan GAIA 3 FIB/SEM, located at the electron microscopy facility (Ce.M.E.) of the National Research Council (CNR) in Florence. For the imaging, we employed a low energy/low flux beam setup, in order to minimize NP damage and ageing. Prior to image acquisition, we deposited the powders on a thin layer of acrylic glue directly deposited on an aluminium stab. We coated the stab with Au (<1 nm thick) by means of sputtering in high vacuum.

1.2 XRD

The phase determination of the synthetic products was carried out using powder XRD, performed using a PANalytical X'PERT PRO powder diffractometer and employing Ni-filtered Cu Kα (1.54187 Å) radiation. We registered the XRD patterns at 1600 W (i.e. 40 kV, 40 mA) with a PIXcel detector and a parabolic MPD mirror. The samples prepared on a silicon wafer (zero-background sample holder) was rotated (2s per rotation) during spectrum acquisition. We recorded the XRD spectrum in the 2θ range 5–100° (step size of 0.026° and a step counting time of 27.5 s). The XRD data were refined by means of a full-profile Rietveld algorithm, using the "GSAS II" software [1]. We kept free the unit-cell parameter values, the crystallite's size and strain as well as the isotropic Debye-Waller factor for the most abundant crystallographic phase. In case a second crystallographic phase is evident, we included an additional structural model in the refinement fitting only the phase fraction, crystallite's size and strain of this associated phase. The instrumental parameters for the Rietveld refinement have been set to the values reported elsewhere [2] for the same instrument used in the present study.

1.3 EMPA – WDS

The chemical element compositions of the samples were analysed through the Electron Microprobe JEOL JXA-8600 (IGG-CNR research unit in Florence & DST). To this aim, we pelleted, enclosed in epoxy resin and polished the samples. The instrument equips four WDS spectrometers, one EDS detector and it is controlled by the software XMas. The system operates

at 10 nA beam current and 15 kV accelerating voltage, with counting times of 15 s on peak and 5 s on each background position. Data were corrected using the PAP matrix correction [3,4].

The estimation of the Cu/S ratio in this kind of multinary sulphides seems to be a major issue for Energy Dispersion Spectroscopy [5]. Although the WDS set up should minimize this problem, we selected a sample of natural covellite (Sample G43082 at the "Museo di Storia Naturale, Sistema Museale di Ateneo dell'Università degli Studi di Firenze" Butte, Montana, USA) as reference.

The Principal Component Analysis required the transformation of the experimental data by using the log-centred transformation [6–8] to achieve robust and unbiased analysis. Eventually, a partioning of the results clarified the statistical dependence between the concentration of the different elements. The partition dwelled on the variance decreasing criterium of the isometric log-ratio transformation [9–11].

1.4 Raman spectroscopy

Raman analyses have been performed at the "Museo di Storia Naturale, Università degli Studi di Firenze – Sistema Museale di Ateneo" laboratories with a Horiba Jobin-Yvon equipped with a 1800 g/mm single holographic grating. The spectrograph coupled to a He-Ne laser source emitting at 632.8 nm (red-light region). We focused the laser beam on the sample using a x50 objective lens resulting in a laser spot of about 10 μm^2 while the slits and holes have been chosen as the widest ensuring the smallest possible instrumental band profile broadening. In these conditions, the microstructural features of the samples yield the whole broadening of the peaks. In this work the standard for the calibration of the frequency is a polycrystalline Si wafer. The main reference for the positions of the Raman peaks is the RRUFF database[12].

1.5 X-Ray Absorption Spectroscopy

X-ray absorption spectroscopy (XAS) measurements at Cu and Sn *K*-edge (8978.9 and 29200.1 *e*V, respectively) were made at the LISA CRG beamline (BM-08; [13]) at the European Synchrotron Radiation Facility (ESRF, Grenoble, France). In order to keep the maximum total absorption (μ) below 1.5 we prepared pellets with adequate aliquots of the samples dispersed in cellulose. We measured all the samples in transmission mode using a pair of Si[111] flat crystals. Si and Pt coated

focusing mirrors ($E_{cutoff} \approx 16$ and 40 K*e*V, respectively) were used for harmonic rejection. The calibration process proceeds by measuring a reference foil during each sample acquisition. The software ATHENA [14] was used to average multiple spectra. Standard procedures [15] were followed to extract the structural EXAFS signal ($k \cdot \chi(k)$): pre-edge background removal, spline modelling of bare atomic background, edge step normalization using a far above the edge region, and energy calibration. Model atomic clusters centred on the absorber atom were obtained by ATOMS [16]. Theoretical amplitude and phase functions were generated using the FEFF8 code [17]. EXAFS spectra were fitted through the ARTEMIS software [14] in the Fourier-Transform (FT) space.

1.6 Diffuse reflectance spectroscopy

The UV–vis-NIR diffuse reflectance spectrum of the as-synthesized products was measured using an Agilent Cary 300 spectrometer, equipped with a Labsphere PELA-1050 integration sphere. The DRS spectra were registered in the range 250–850 nm, at a step of 1 nm, counting 0.1 s per step. Reflectance of samples was compared with a standard fluoropolymer (Spectralon), characterized as the highest diffuse reflectance of any known material in this region of the spectrum.

Section 2: Experimental

2.1 EMPA – WDS

The longest rays Cl, Zn and Sn quadrants where samples of different groups are well discriminated indicating the high homogeneity of the samples. The long rays of Cl and Sn pointing to almost opposite direction suggest a strong inverse relationship, indicating that they belong to different phases. Moreover, this seems to be the main discriminant between sample S2 and S3. Cu and S point to different directions, suggesting that they could be partially segregated. The biplot confirms that the main source of variability is the Zn amount. Indeed, Zn is involved only in S1 (Tab. 1). The second most important source of variability is Cl (numerator of balance 2 – Tab. 2). Balances 3 and 4 (Tab. 2) regards the species involved in a CTS phases. Balance 3 (Tab. 2) shows that Sn is a consistent source of variability. Balance 4 is the last term of the data reduction process.

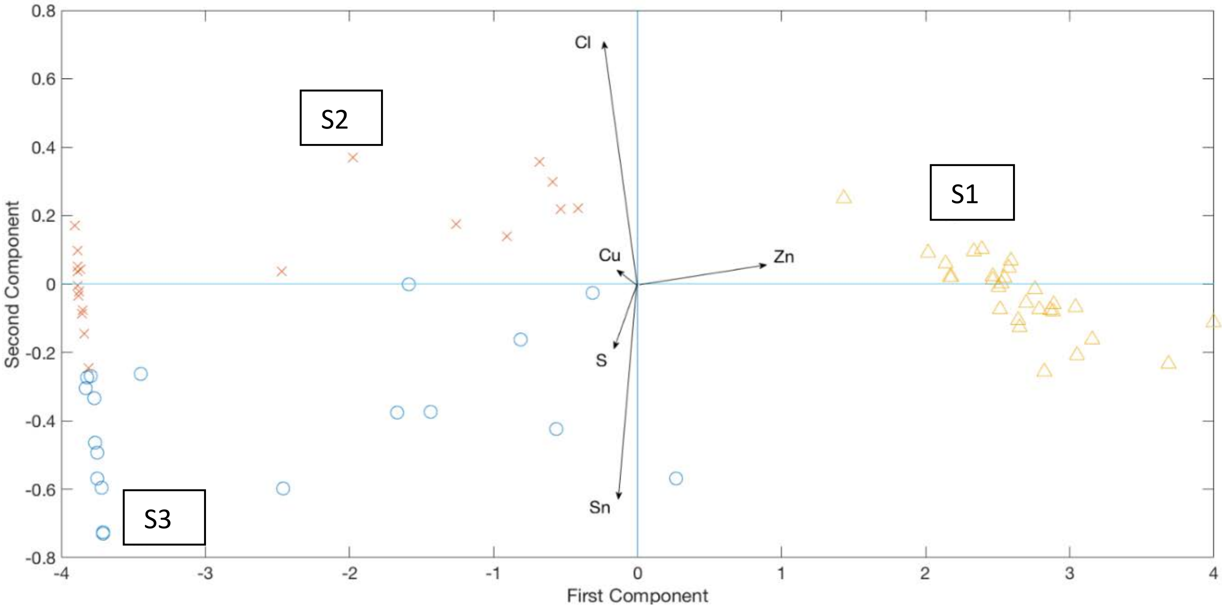

Figure S1 biplots of the EMPA compositional data.

Figure S2 presents the relationship between these balances in different samples. There is nearly no dependence between balance 2 and 1 (Figure S2a) for none of the samples. This suggests that Cl is segregated in associated phases (unreacted species). Conversely, the dependence between balance 3 and 1, as well as the dependence between balance 4 and 1 suggests a relationship between

the amounts of Sn, S and Zn in the sample S1 (Figures S2b,c). In Figure S1d the inverse trend between balance 3 and balance 2 reveals that the amounts of Sn and Cl are inversely correlated: this could be attributed to the fact that they are likely segregated in different phases. Similarly, in Figure S1e the inverse trend between balance 4 and balance 2 reveals that the amounts of S and Cl are inversely correlated (meaning that there is correlation between Cu and Cl). Hence, Cu and Cl are likely to be partially co-loacalized in an associated phase. Eventually, the strong dependence between balance 4 and balance 3 in figure S2f reveals that Sn and S are strongly correlated, hence they are mostly present in the same phase. Conversely, Cu can be partially separated by Sn and S in a associated phase. Moreover, figure S2f shows also that the datapoints of sample S2 and S1 cluster around the position of kuramite in the transformed space suggesting that S1 and S3 are homogeneously consistent with the composition of kuramite.

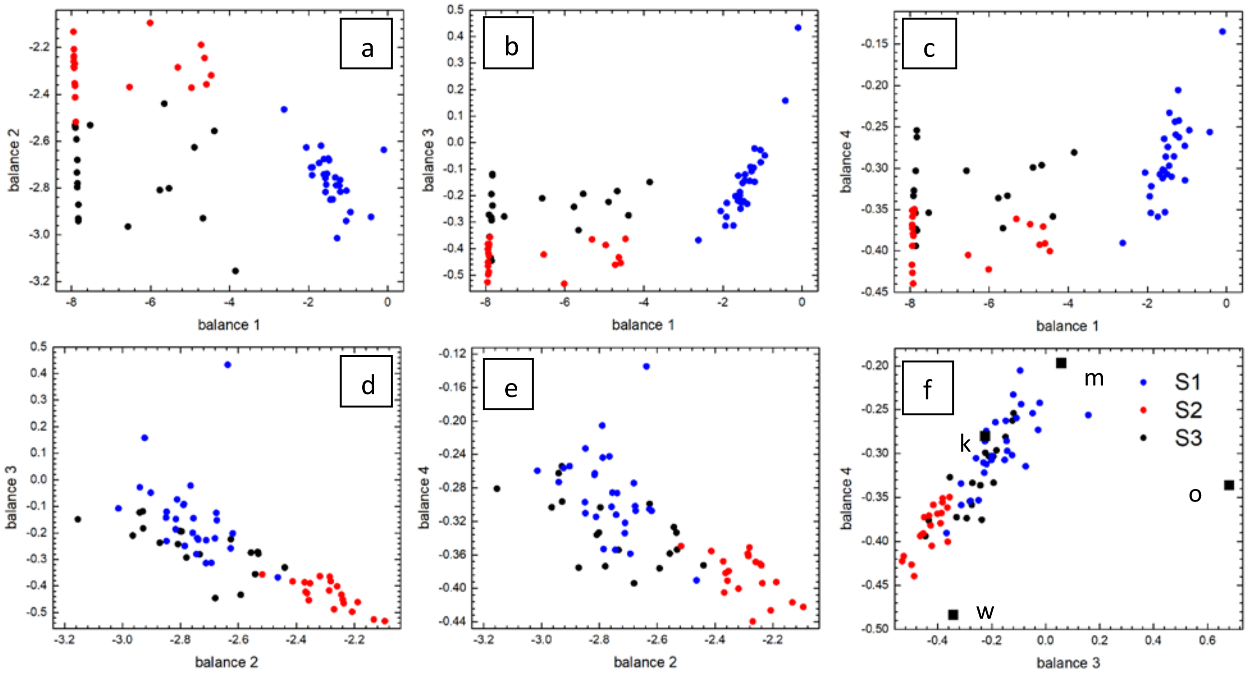

Figure S2 Plot of the trends between two difference balances, where the squares mark the position of the considered phases in the transformed space k) Cu_3SnS_4, m) Cu_2SnS_3, w) Cu_4SnS_4 and o) $Cu_4Sn_7S_{16}$.

2.2 XRD

The tetragonal model of the CTS structure is 2a supercell of a sphalerite-type subunit. For the sample S2 the refined diffractogram involves a bi-phasic model including tetragonal CTS and covellite (JCPDS 06-0464 [18]) as associated phase. The refined lattice (Tab. S1) parameters compare very well with those of kuramite (a = 5.445 Å; c = 10.75 Å; JCPDS 33-0501 [19]) or mohite (ICDD PDF 04-010-5719; a = 5.413 Å; c = 10.82 Å; JCPDS [20]). We fitted the **a** lattice parameter of the cubic cell and we compared it with the average of the lattice parameters of the tetragonal kuramite (JCPDS 33-0501; Kovalenker, 1981) and mohite (JCPDS ; X. Chen et al., 1998) according to the following formula (see also Figure S3):

$$a_{cubic} = \frac{a_{tetragonal} + b_{tetragonal} + \frac{c_{tetragonal}}{2}}{3}$$

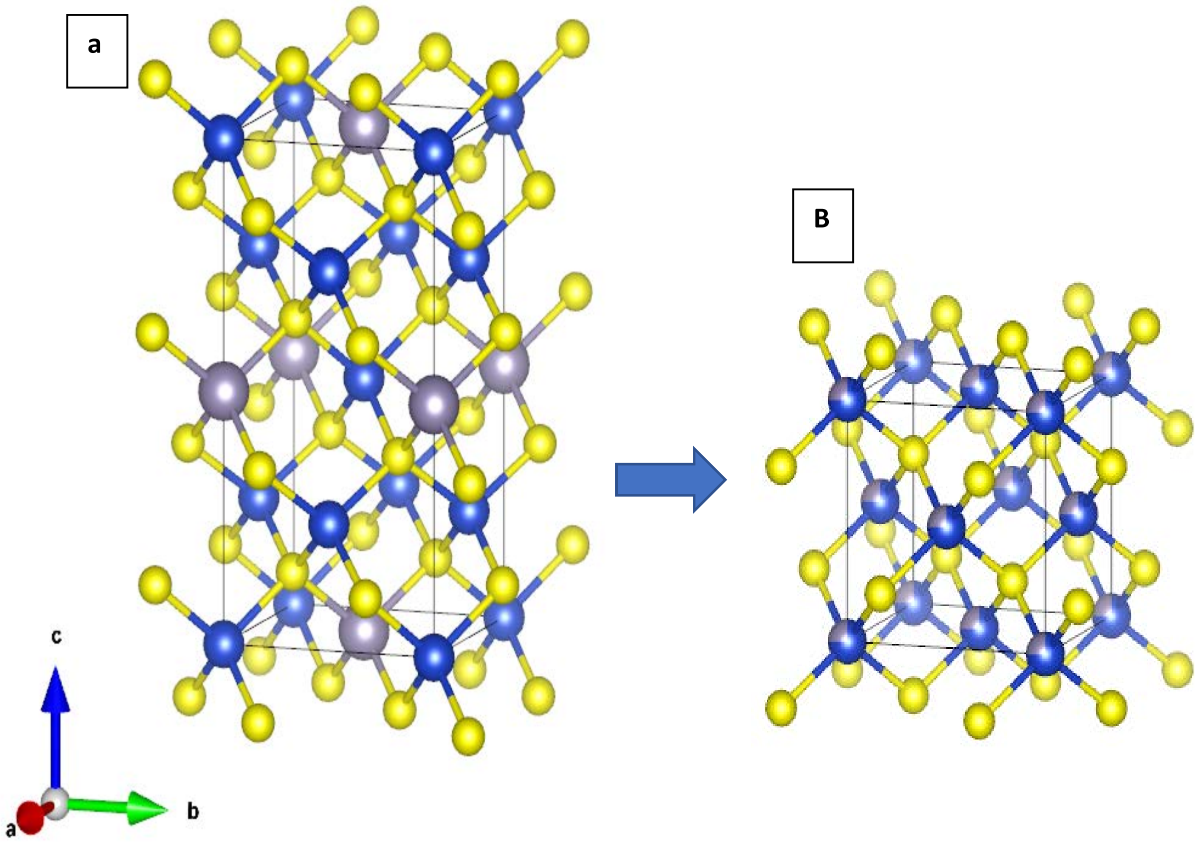

Figure S3 The two kuramite's model a) tetragonal model ($I\bar{4}2m$) b) derived cubic model ($F\bar{4}3m$).

Table S1 Best fits of the diffractograms with the tetragonal CTS structural model compared with the tetragonal structural models derived in this paper. The acronym CV refers to covellite.

$I\bar{4}\,2\,m$	a(Å)	c(Å)	vol(Å^3)	Rf%	Size (nm)	% CV	Rf% CV	Ref.
**S3**	5.439(3)	10.712(5)	316(1)	2.48%	9	N/D	N/D	This study
**Δ% kuramite**	-0.11%	-0.38%	-0.60%					
**Δ% mohite**	0.48%	-1.1	-0.11%					
**S2**	5.431(2)	10.750(6)	317(1)	2.90%	10	28%	3.54%	This study
**Δ% kuramite**	-0.26%	-0.004%	-0.51%					
**Δ% mohite**	0.33%	-0.68%	-0.014%					
**S1**	5.444(1)	10.714(5)	317.5(7)	2.57%	9	N/D	N/D	This study
**Δ% kuramite**	-0.013%	-0.35%	-0.37%					
**Δ% mohite**	0.58%	-1.03%	0.12%					
**kuramite**	**5.445**	**10.75**	**318.72**					19
**mohite**	**5.413**	**10.824**	**317.149**					20

As shown by Table S2, the cubic structural model yields better results even if involving a lower number of free parameters (3 Debye-Waller factors for the cubic CTS and 4 for the tetragonal CTS).

Any attempt to refine the diffractograms relaxing the preferential orientation along the crystallographic direction corresponding to the most intense peak ([111] for the cubic CTS and [220] for tetragonal CTS) lead to an insignificant improvement of the Rf%. Moreover, the refinement process yields values of the March-Dollase parameter not significantly different from 1. Clearly, we can deduce that no preferential orientation can be reckoned.

Table S2 Best fits of the diffractograms with the cubic CTS structural model compared with the cubic structural models derived in this paper. The acronym CV refers to covellite.

$F\bar{4}3m$	a(Å)	Vol(Å^3)	Rf%	Size (nm)	%CV	Rf% CV	Ref
**S3**	5.432(3)	160.3(9)					
**Δ% cubic Cu_3SnS_4**	0.19%	0.57%	2.11%	13	N/D	N/D	This study
**Δ% cubic Cu_2SnS_3**	0.36	1.07%					
**S2**	5.413(4)	158(1)					
**Δ% cubic Cu_3SnS_4**	-0.17%	-0.50%	1.46%	9	23%	4.73%	This study
**Δ% cubic Cu_2SnS_3**	-0.0029%	-0.0086%					
**S1**	5.417(2)	158.9(8)					
**Δ% cubic Cu_3SnS_4**	-0.093%	-0.28%	1.69%	12	N/D	N/D	This study
**Δ% cubic Cu_2SnS_3**	0.073%	0.22%					
**Cubic Cu_3SnS_4**	**5.422**	**159.4**					**This study**
**Cubic Cu_2SnS_3**	**5.413**	**158.6**					**This study**

2.3 Raman

The fit of sample S2 shows a component at 350 cm^{-1} which is thinner with respect to the other two components, thus it could be attributed to different phase than the main in sample S2 (which resemble closely S1 and S3). Indeed, the attribution of this peak is not straightforward, it is not chalcocite, covellite (found by XRD), berndite, herzenbergite nor CuCl, $CuCl_2$, $SnCl_2$ or $SnCl_4$. It could be another phase of Cu_3SnS_4 or Cu_2SnS_3[21] but there are not enough evidences to support it.

Table S3 Best fits of the most intense Raman peaks of the spectra as shown in Figure 5.

	1st component			2nd component			3rd component		
	Position (A.U.)	Area (A.U.)	Width (nm)	Position (A.U.)	Area (A.U.)	Width (nm)	Position (A.U.)	Area (A.U.)	Width (nm)
**S1**	282.4(3)	6.1(2) 10^3	42(1)	330.8(1)	1.81(2) 10^4	48.3(4)			
**S2**	286.5(5)	9.99(3) 10^3	53(1)	327.9(3)	1.35(7) 10^4	34(1)	347.6(4)	3.2(4) 10^3	22(1)
**S3**	284.5(5)	1.01(3) 10^4	56(1)	332.4(2)	1.40(3) 10^4	44.0(6)			

The total phonons at the Γ point in the Brillouin zone corresponds to the coordinates of the atoms in the Bravais cell. Hence, 24 phonons for the tetragonal Cu_3SnS_4 ($I\bar{4}2m$) and 6 for the cubic Cu_3SnS_4 ($F\bar{4}3m$). On this basis, the factor group analysis yields 3 non-translational normal modes for the cubic Cu_3SnS_4 ($F\bar{4}3m$) and 5 for the tetragonal Cu_3SnS_4 ($I\bar{4}2m$).

Table S4 Result of the factor group analysis of the phonons at the Γ point in the brillouin zone.

	Decomposition of the *rep*	Raman active mode	Ref
**Tetragonal Cu_3SnS_4**	$\Gamma_{121} = 2\Gamma_1 + \Gamma_2 + 2\Gamma_3 + 5\Gamma_4 + 7\Gamma_5$	$\Gamma_1, \Gamma_3, \Gamma_4, \Gamma_5$	(This study)
**Cubic Cu_3SnS_4**	$\Gamma_{216} = 2\Gamma_5$	Γ_5	(This study)

2.4 Diffuse reflectance spectroscopy

The Tauc plot (Fig. S4)[22] is reported only for sample S3 which is the cleanest kuramite sample. The extrapolation of the band gap is carried out on the linear part of the $(A\cdot E)^2$ function [23,24]. The value, 1.6 eV compares well with the data reported in the literature [25] for microcrystalline. Discrepancies in the range of hundreds meV between the ideal and experimental value of the band gap is due to effect of tail states (or Urbach tails) [26,27]. The results here obtained are fully compatible with the optimal range for solar energy conversion (i.e., photovoltaic and photocatalytic applications).

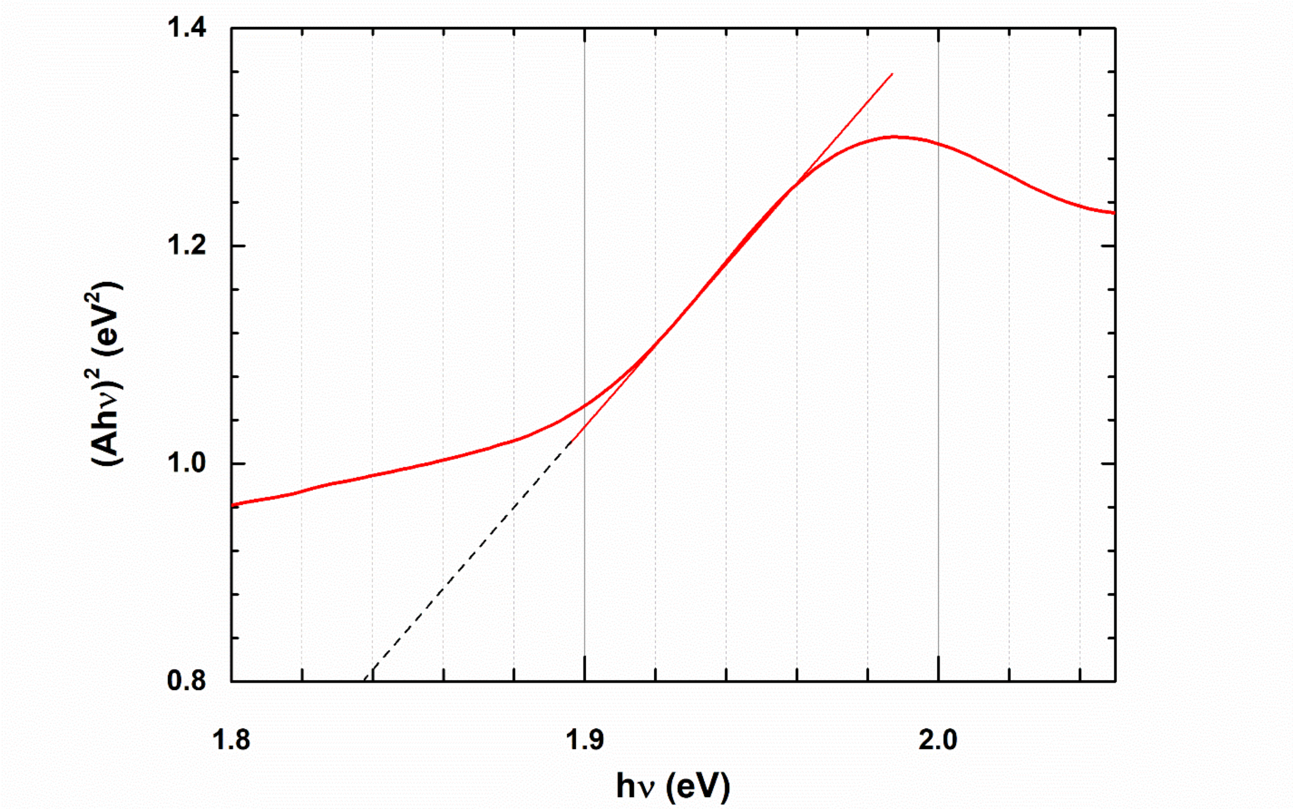

Figure S4 Tuac plot with the linear extrapolation of the band gap energy for sample S3.

Table S5 Results of the recalculation of the composition of sample S2 after subtracting both the estimated amount of $CuCl_2$ and CuS. Comparison with the raw composition (apfu (raw)) and the composition of the ideal phases.

	**apfu (raw)**	**apfu**	**Mohite**	**kuramite**	**Wang**
**S2**					
**S**	3.96(4)	3.82(4)	4.00	**4.00**	3.56
**Cu**	3.23(4)	3.12(4)	2.67	**3.00**	3.56
**Sn**	0.81(1)	1.04(1)	1.33	**1.00**	0.889

References

(1) Toby, B. H.; Von Dreele, R. B. *GSAS-II* : The Genesis of a Modern Open-Source All Purpose Crystallography Software Package. *J. Appl. Crystallogr.* **2013**, *46* (2), 544–549. https://doi.org/10.1107/S0021889813003531.

(2) Kaduk, J. A.; Reid, J. Typical Values of Rietveld Instrument Profile Coefficients. *Powder Diffr.* **2011**, *26* (01), 88–93. https://doi.org/10.1154/1.3548128.

(3) Bastin, G. F.; Heijligers, H. J. M. Quantitative Electron Probe Microanalysis of Ultra-Light Elements. *J. Microsc. Spectrosc. électroniques* **1986**, *22*, 215–228.

(4) Pouchou JL, P. F. Basic Expressions of PAP Computation for Quantitative EPMA. In *Proceedings of ICXOM 11, Ontario*; 1987; pp 249–253.

(5) Giaccherini, A.; Baldassarre, A.; Donini, L.; Lepore, G. O.; Caneschi, A.; De Luca, A.; Innocenti, M.; Montegrossi, G.; Giuseppe, C.; Oberhauser, W.; et al. Sustainable Synthesis of Quaternary Sulphides: The Problem of the Uptake of Zinc in CZTS. *J. Alloys Compd.* **2019**, *775*, 1221–1229. https://doi.org/10.1016/J.JALLCOM.2018.10.201.

(6) Society, R. S. The Statistical Analysis of Compositional Data Author (s): J . Aitchison Source : Journal of the Royal Statistical Society . Series B (Methodological), Vol . 44 , No . 2 Published by : Wiley for the Royal Statistical Society Stable URL : Https://Www.J. **2018**, *44* (2), 139–177.

(7) Varmuza, K.; Filzmoser, P. *Introduction to Multivariate Statistical Analysis in Chemometrics*, 1st editio.; CRC Press, Ed.; 2009.

(8) Buccianti, A.; Grunsky, E. Compositional Data Analysis in Geochemistry: Are We Sure to See What Really Occurs during Natural Processes? *J. Geochemical Explor.* **2014**, *141*, 1–5. https://doi.org/10.1016/j.gexplo.2014.03.022.

(9) Egozcue, J. J.; Pawlowsky-Glahn, V. Groups of Parts and Their Balances in Compositional Data Analysis. *Math. Geol.* **2005**, *37* (7), 795–828. https://doi.org/10.1007/s11004-005-7381-9.

(10) Egozcue, J. J.; Pawlowsky-Glahn, V.; Mateu-Figueras, G.; Barceló-Vidal, C. Isometric Logratio Transformations for Compositional Data Analysis. *Math. Geol.* **2003**, *35* (3), 279–300. https://doi.org/10.1023/A:1023818214614.

(11) Martín-Fernández, J. A.; Pawlowsky-Glahn, V.; Egozcue, J. J.; Tolosona-Delgado, R. Advances in Principal Balances for Compositional Data. *Math. Geosci.* **2018**, *50* (3), 273–298. https://doi.org/10.1007/s11004-017-9712-z.

(12) Lafuente, B.; Downs, R. T.; Yang, H.; Stone, N. *The Power of Databases: The RRUFF Project*; 2016. https://doi.org/10.1515/9783110417104-003.

(13) D'Acapito, F.; Trapananti, A.; Puri, A. LISA: The Italian CRG Beamline for x-Ray Absorption Spectroscopy at ESRF. *J. Phys. Conf. Ser.* **2016**, *712* (1), 2–6. https://doi.org/10.1088/1742-6596/712/1/012021.

(14) Ravel, B.; Newville, M. ATHENA, ARTEMIS, HEPHAESTUS: Data Analysis for X-Ray Absorption Spectroscopy Using IFEFFIT. *J. Synchrotron Radiat.* **2005**, *12* (4), 537–541. https://doi.org/10.1107/S0909049505012719.

(15) Lee, P. A.; Citrin, P. H.; Eisenberger, P.; Kincaid, B. M. Extended X-Ray Absorption Fine Structure—Its Strengths and Limitations as a Structural Tool. *Rev. Mod. Phys.* **1981**, *53*, 769.

(16) Ravel, B. ATOMS: Crystallography for the X-Ray Absorption Spectroscopist. *J. Synchrotron Radiat.* **2001**, *8* (2), 314–316. https://doi.org/10.1107/S090904950001493X.

(17) Ankudinov, A. L.; Ravel, B.; Rehr, J. J.; Conradson, S. D. Real-Space Multiple-Scattering Calculation and Interpretation of x-Ray-Absorption near-Edge Structure. *Phys. Rev. B* **1998**, *58* (12), 7565–7576.

(18) Howard T. Evans, J.; Konnert, J. A. Crystal Structure Refinement of Covellite. *Am. Mineral.* **1976**, *61*, 996–1000.

(19) Kovalenker, V. A. Kuramite, Cu_3SnS_4, a New Mineral of the Stannite Group. *Int. Geol. Rev.* **1981**, *23* (3), 365–370. https://doi.org/10.1080/00206818109455070.

(20) Chen, X.; Wada, H.; Sato, A.; Mieno, M. Synthesis, Electrical Conductivity, and Crystal Structure of Cu4Sn7S16and Structure Refinement of Cu2SnS3. *J. Solid State Chem.* **1998**, *139* (1), 144–151. https://doi.org/10.1006/jssc.1998.7822.

(21) Raadik, T.; Grossberg, M.; Krustok, J.; Kauk-Kuusik, M.; Crovetto, A.; Bolt Ettlinger, R.; Hansen, O.; Schou, J. Temperature Dependent Photoreflectance Study of Cu_2SnS_3 Thin Films Produced by Pulsed Laser Deposition. *Appl. Phys. Lett.* **2017**, *110* (26), 261105. https://doi.org/10.1063/1.4990657.

(22) J. Tauc, R. G. and A. V. Optical Properties and Electronic Structure of Amorphous Germanium. *Phys. status solidi b* **1966**, *15* (2), 627–637.

(23) Boldish, S. I.; White, W. B. Optical Band Gaps of Selected Ternary Sulfide Minerals. *Am. Mineral.* **1998**, *83* (7–8), 865–871.

(24) Adelifard, M.; Eshghi, H.; Bagheri Mohagheghi, M. M. Synthesis and Characterization of Nanostructural CuS-ZnS Binary Compound Thin Films Prepared by Spray Pyrolysis. *Opt. Commun.* **2012**, *285* (21–22), 4400–4404. https://doi.org/10.1016/j.optcom.2012.06.030.

(25) Botti, S.; Kammerlander, D.; Marques, M. A. L. Band Structures of Cu2ZnSnS4 and Cu2ZnSnSe4 from Many-Body Methods. *Appl. Phys. Lett.* **2011**, *98* (24), 241915. https://doi.org/10.1063/1.3600060.

(26) Siebentritt, S.; Rey, G.; Finger, A.; Regesch, D.; Sendler, J.; Weiss, T. P.; Bertram, T. What Is the Bandgap of Kesterite? *Sol. Energy Mater. Sol. Cells* **2016**, *158*, 126–129. https://doi.org/10.1016/j.solmat.2015.10.017.

(27) Scragg, J. J. S.; Larsen, J. K.; Kumar, M.; Persson, C.; Sendler, J.; Siebentritt, S.; Platzer Björkman, C. Cu-Zn Disorder and Band Gap Fluctuations in $Cu_2ZnSn(S,Se)_4$: Theoretical and Experimental Investigations. *Phys. status solidi* **2016**, *253* (2), 247–254. https://doi.org/10.1002/pssb.201552530.
